# Hydroxychloroquine reduces soluble Flt-1 secretion from human cytotrophoblast, but does not mitigate markers of endothelial dysfunction in vitro

**DOI:** 10.1371/journal.pone.0271560

**Published:** 2022-11-23

**Authors:** Elif Kadife, Natalie Hannan, Alesia Harper, Natalie Binder, Sally Beard, Fiona C. Brownfoot

**Affiliations:** 1 Department of Obstetrics and Gynaecology, Obstetric Diagnostics and Therapeutics Group, University of Melbourne, Melbourne, Victoria, Australia; 2 Mercy Perinatal, Mercy Hospital for Women, Melbourne, Victoria, Australia; 3 Department of Obstetrics and Gynaecology, Therapeutics Discovery and Vascular Function in Pregnancy, University of Melbourne, Melbourne, Victoria, Australia; Universita degli Studi di Padova, ITALY

## Abstract

Preeclampsia is a multi-system disease that can have severe, even fatal implications for the mother and fetus. Abnormal placentation can lead to ischaemic tissue injury and placental inflammation. In turn, the placenta releases anti-angiogenic factors into the maternal circulation. These systemically act to neutralise angiogenic factors causing endothelial dysfunction causing preeclampsia. Hydroxychloroquine is an immune modulating drug that is considered safe in pregnancy. There is epidemiological evidence suggesting it may reduce the risk of preeclampsia. Here, we examined the effects hydroxychloroquine on the production and secretion of sFlt-1, soluble endoglin (sENG), placental growth factor (PlGF) and vascular endothelial growth factor (VEGF) in primary human placenta, cytotrophoblasts and umbilical vein endothelial cells (endothelial cell model). Hydroxychloroquine treatment decreased mRNA expression of two sFlt-1 isoforms and its protein secretion. sENG was not reduced. Hydroxychloroquine treatment increased secretion of pro-angiogenic factor PIGF from endothelial cells. It did not significantly reduce the expression of the endothelial cell inflammation marker, *ET-1*, and inflammation induced expression of the adhesion molecule, *VCAM*. Hydroxychloroquine could not overcome leukocyte adhesion to endothelial cells. Hydroxychloroquine mitigates features of preeclampsia, but it does not reduce key markers of endothelial dysfunction.

## Introduction

Preeclampsia complicates approximately 5% of pregnancies worldwide and is a leading cause of maternal and perinatal morbidity and mortality [1, 2]. Clinically, it is defined as hypertension during pregnancy accompanied by uteroplacental insufficiency, proteinuria, or major organ dysfunction [3]. The only treatment is delivery. If preterm birth is required, it can inflict a lifetime risk of comorbidities arising from prematurity, on the baby.

Preeclampsia is thought to arise from abnormal placentation early in pregnancy, leading to inadequate invasion and remodelling of the maternal spiral arterioles. As the placenta grows it likely outstrips its blood supply and inflammation, ischemia and hypoxia ensue [4, 5]. This results in the secretion of anti-angiogenic factors, soluble fms-like tyrosine (sFlt-1) and soluble endoglin (sENG), that neutralise the activity and reduce the biological action of pro-angiogenic molecules, placental growth factor (PIGF) and vascular endothelial growth factor (VEGF) [6]. This imbalance contributes to systemic endothelial dysfunction characterised by weakened cellular junctions, increased vascular permeability and reduced capacity for endothelium-dependent vasodilation [7]. Thus, the anti-angiogenic, inflammatory and hypoxic environment of the preeclamptic placenta form a destructive loop, each factor reinforcing and aggravating the other.

There is epidemiological evidence to suggest hydroxychloroquine may reduce the incidence of preeclampsia in patients with autoimmune conditions [8]. Furthermore, hydroxychloroquine was shown to rescue tight junction loss in human umbilical vein derived endothelial cells (HUVECs), mediated by tumour necrosis factor-α (TNFα) and preeclampsia sera treatments, *in vitro* [9]. Hydroxychloroquine has immunomodulatory and antioxidant effects [10]. Its main mode of action is to inhibit hydrolytic enzymes within lysosomes and endosomes, thus preventing the degradation of the content in their cargos and ultimately suppressing the presentation of self-antigens to immune cells. This results in a reduction in the activation of pattern-recognition receptors, such as toll-like receptors. These receptors are part of the innate immune system: they sense damage associated molecular patterns in the form of lipids, proteins or nucleic acid and activate inflammation [11]. When these receptors are activated, they trigger nuclear factor- kappa beta mediated release of pro-inflammatory cytokines like TNFα and interleukin-6 [12]. These factors and cytokines are also highly active in preeclamptic placentas and likely contribute to the progression of the disease. Importantly, several clinical studies have demonstrated that hydroxychloroquine posed no increased risk in pregnancy related complications in women with auto-immune conditions, albeit a higher observation of preterm births that was attributed to underlying disease activity, rather than the drug [13–15]. Furthermore, hydroxychloroquine was not found to pose a serious risk to congenital and developmental progress.

Whilst there are various clinical studies measuring hydroxychloroquine mediated outcomes in patients with auto-immune conditions, functional preeclampsia focused studies utilising different cell population of the placenta are limited. We address this by using trophoblasts, human umbilical vein endothelial cells and explants. Furthermore, there are no studies reporting the effect of hydroxychloroquine on biomarkers of preeclampsia. As such, in this study we aimed to determine whether hydroxychloroquine treatment affects transcription and secretion of anti-angiogenic and/or pro-angiogenic molecules *in vitro*. Additionally, as another hallmark of preeclampsia, we also considered the impact of hydroxychloroquine on endothelial cell dysfunction not just by evaluating the expression of markers but also in functional assays.

## Materials and methods

To characterise effects of hydroxychloroquine on anti-angiogenic markers of preeclampsia and endothelial dysfunction, we isolated human cytotrophoblast, as well as tissue explants and HUVECs from term human placenta. Secretion and expression of biomarkers associated with preeclampsia including PIGF, VEGF, sFlt-1 and sENG. We also examined markers of endothelial dysfunction endothelin 1 (*ET1*) and vascular cell adhesion marker (*VCAM*).

### Isolating and treating primary human cytotrophoblast cells

Term placenta cytotrophoblasts (n = 3) were isolated from women having elective caesarean sections as described previously [16]. Cells were seeded at 24,000 cells /cm^2^ density and treated with 0, 1, 2, 5, 10, 20 μM of hydroxychloroquine (Sigma) for 24 hours at 37°C in 8% O_2_ and 5% CO_2_ (n = 3). Cell lysates and conditioned media were collected from these cultures. ELISA assays were used to determine sFlt-1 and sENG concentration in the media and the expression of sFlt-1 splice variants *sFlt-1 e15a* and *sFlt-1 i13* were evaluated using qPCR.

### Culture of placental explants

Placental explants (n = 5) were collected from women having an elective caesarean section at term and were prepared as previously described [16]. After we cultured the explants in media for 24 hours, they were treated with hydroxychloroquine (0, 1, 2, 5, 10, 20 μM) for 48 hours at 37°C, 8% O_2_ and 5% CO_2_. Tissue was collected and weighed, and RNA was extracted for qPCR to determine *VEGF* and *PIGF* expression. Conditioned media was collected to determine secreted sFlt-1 and sENG protein levels using ELISA.

### Isolating and treating primary human umbilical vein endothelial cells (HUVECs)

We isolated HUVECs from normal term placentas as previously published [16]. Cells were plated at a density of 24,000 cells /cm^2^ and treated with 0, 1, 2, 5, 20μM of hydroxychloroquine (Sigma) n = 3 for 24 hours at 37°C in 20% O_2_ and 5% CO_2_. Cell lysates were collected for RNA and protein analysis and conditioned media was collected for examination of secreted factors. The media was screened for soluble factor PIGF, while expression of *PIGF*, *VEGF*, *VCAM*, *ET1* were investigated using qPCR.

### Quantitative Polymerase Chain Reaction (qPCR)

Extraction of RNA from placental cytotrophoblasts, explants and HUVECs were performed with the RNeasy mini kit (Qiagen, Valencia, CA) according to manufacturer’s instructions and quantified using the Nanodrop ND 1000 spectrophotometer (NanoDrop technologies Inc, Wilmington, DE). After this 0.2 μg of RNA was converted to cDNA using the Applied Biosystems high-capacity cDNA reverse transcriptase kit (Life Technologies) in line with the manufacturer’s guidelines.

We assessed gene expressions of *VCAM-1* (Life Technologies), *PlGF* (Life Technologies) by real time PCR (RT-PCR) on the CFX 384 (Bio-Rad, Hercules, CA) using FAM-labeled Taqman universal PCR mastermix and its specific primer/probe set (Life Technologies) with the following run conditions: 50°C for 2 minutes; 95°C for 10 minutes, 95°C for 15 seconds, 60°C for 1 minute (40 cycles). The data was normalised to housekeeper genes *YHWAZ* and calibrated against the average C_t_ of the control samples. Results were expressed as percentage fold change from control.

SYBR RT-PCR was carried out to assess gene expressions of *sFlt-1 e15a* and *sFlt-1 i13* and *GAPDH*. We designed primers as previously described (Geneworks, South Australia, Australia). We performed quantitative RT-PCR using the run conditions: 95°C for 20 minutes; 95°C for 0.01 minutes, 60°C for 20 minutes, 95°C for 1 minute (39 cycles), melt curve 65°C to 95°C at 0.05°C increments at 0.05 seconds. The data was normalised to housekeeper genes *GAPDH*, *Topo or Cyt-c* and calibrated against the average C_t_ of the control samples.

### ELISA analysis

sFlt-1, sENG and PlGF concentrations were measured in the conditioned media using the DuoSet VEGF R1/Flt-1 kit (R&D systems by Bioscience, Waterloo, Australia), a DuoSet Human Endoglin CD/105 ELISA kit (R&D systems) or Duoset Human PlGF kit (R&D systems). We followed the manufacturer’s instructions.

### Endothelial dysfunction in primary HUVECs

To induce endothelial cell dysfunction, the isolated primary HUVECs (n = 4) were stimulated with 1ng/ml TNFα. After 24 hours, increasing doses of hydroxychloroquine (1, 2, 5, 20μM) were added to the cultures and the cells were treated for a further 24 hours. *VCAM* and *ET1* mRNA expression were measured by qPCR.

### Adhesion assay

Primary HUVECs (n = 5) were treated with 1 ng/ml TNFα alone or in combination with 5 and 20μM doses of hydroxychloroquine for 24 hours at 20% O2, 5% CO2 and 37°C. For peripheral blood monocyte (PBMC) isolations, blood samples were obtained from pregnant patients in EDTA vacutainers, then centrifuged to separate out and discard plasma layer, leaving behind cells. The cells were then diluted in PBS and fractionated using 12mL Ficoll-Paque (GE Healthcare, Little Chalfont, UK) and centrifuged at 400×g for 30 min without brakes. The PBMCs were collected, washed, and centrifuged again at 300×g for 10 min to remove any remaining red blood cells, which were lysed before the isolated PBMCs were stained with fluorescent calcein dye (Merck Millipore, Darmstadt, Germany) for 30 min at 37°C. After this they were co-cultured with HUVECs for 45 minutes. Unattached PBMCs were removed, cells were gently washed, and the fluorescent intensity of the adhesion was detected and quantified using FLUOstar OMEGA fluorescent plate reader (BMG labtech, Ortenberg, Germany) at 485/520 nm.

### Ethics approval

Ethics approval was provided by The Mercy Health Human Research Ethics Committee (Institutional review board number R11/34 and R14/11) and written consent was obtained from all participants.

### Statistical analysis

All experiments were performed with a minimum of three technical triplicates for each biological replicate and there were at least three patients for each experiment (three biological replicates). Statistical analysis was conducted using a t-test (parametric) or a Mann-Whitney test (non-parametric) for two groups and a one-way ANOVA (parametric) or a Kruskal-Wallis test (non-parametric) where there were three or more groups being compared. We used the GraphPad Prism 6 (GraphPad Software, La Jolla, CA) for statistical analysis. All data were expressed as mean ± SEM; P values < 0.05 were considered significant.

## Results

### Effect of hydroxychloroquine on sFlt-1 expression and secretion from cytotrophoblasts and explants

**Fig 1:**
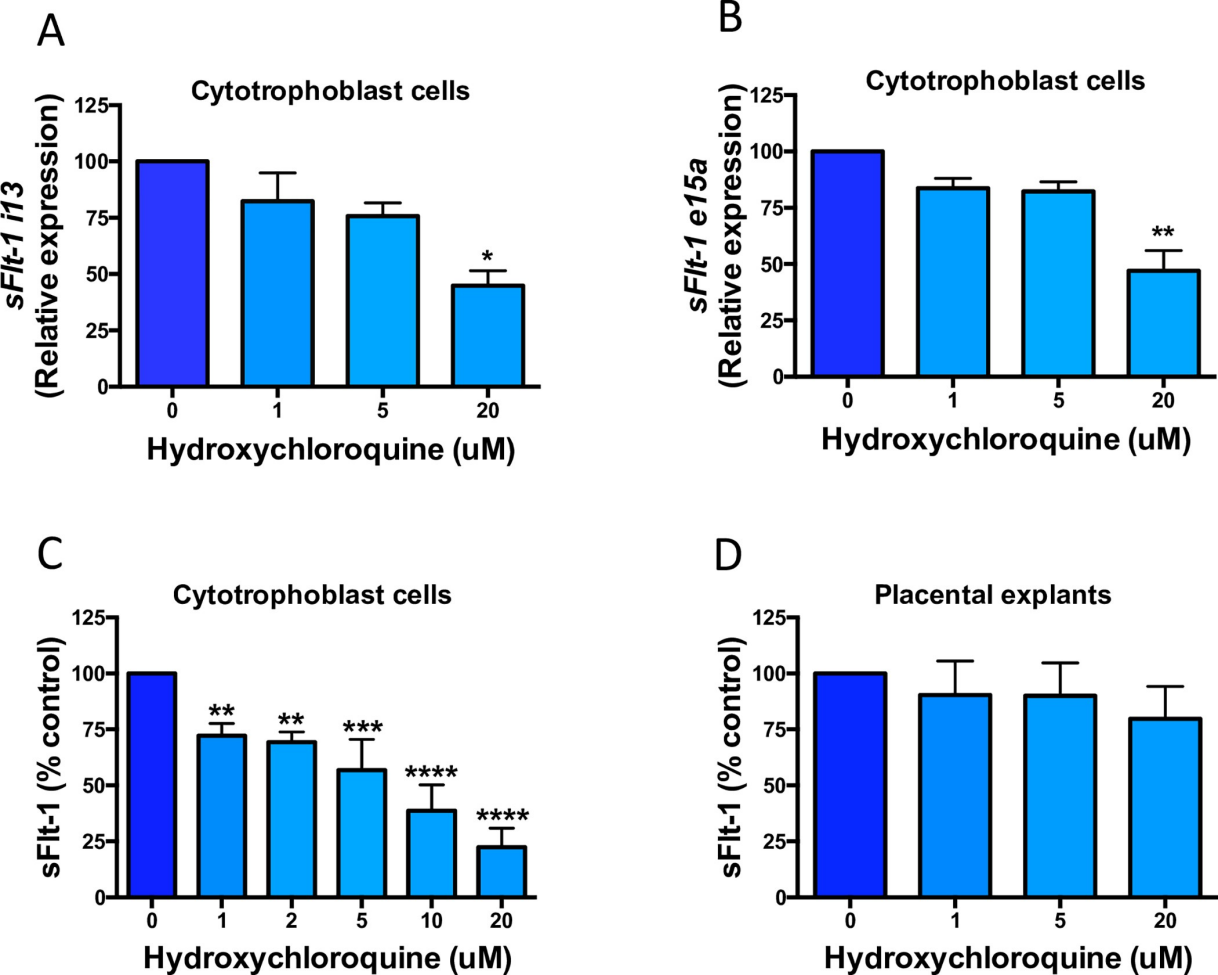
The effects of hydroxychloroquine on sFlt-1 gene expression and its secretion from cytotrophoblasts and explants of preeclamptic placentas. Treating cytotrophoblasts with hydroxychloroquine caused a significant reduction in both sFlt-1 variant isoforms *sFlt-1 i13* (a) and *sFlt-1 e15a* (b) with a 20μM dose of hydroxychloroquine. This reduction translates into a significant decrease of sFlt-1 secretion from cytotrophoblasts (c) across all doses of the drug. Although not statistically significant, on average placental explants tended produce less sFlt-1 under treatment. Data are means ± SEM of minimum three independent experiments. * p<0.05, ** p<0.01, *** p<0.001, **** p<0.0001 (Friedman test).

Treating the cells and explants with increasing doses of hydroxychloroquine (1–20μM), we examined its effect on the expression of sFlt-1 splice variants (*e15a* and *i13*). The lower doses (1μM and 5μM) slightly decreased expression of *sFlt-1 i13* and *sFlt-1 e15a* in cytotrophoblasts, however, on average the 20μM dose led to a 50% reduction in these genes (Fig 1A and 1B). Nonetheless, even small decreases in gene expression with drug treatment appears to significantly reduce secretion of sFlt-1 from these cells. In a dose dependent manner, the conditioned media from treated cells showed 25–75% lower sFlt-1, compared to vehicle control cells (Fig 1C). A trend towards decreased sFlt-1 concentrations was observed in conditioned media from treated explant tissue, however, this did not reach statistical significance (Fig 1D). Thus, hydroxychloroquine downregulates gene and protein expression of sFlt-1.

### Effect of hydroxychloroquine on sENG secretion from primary endothelial cells and placental tissue

**Fig 2:**
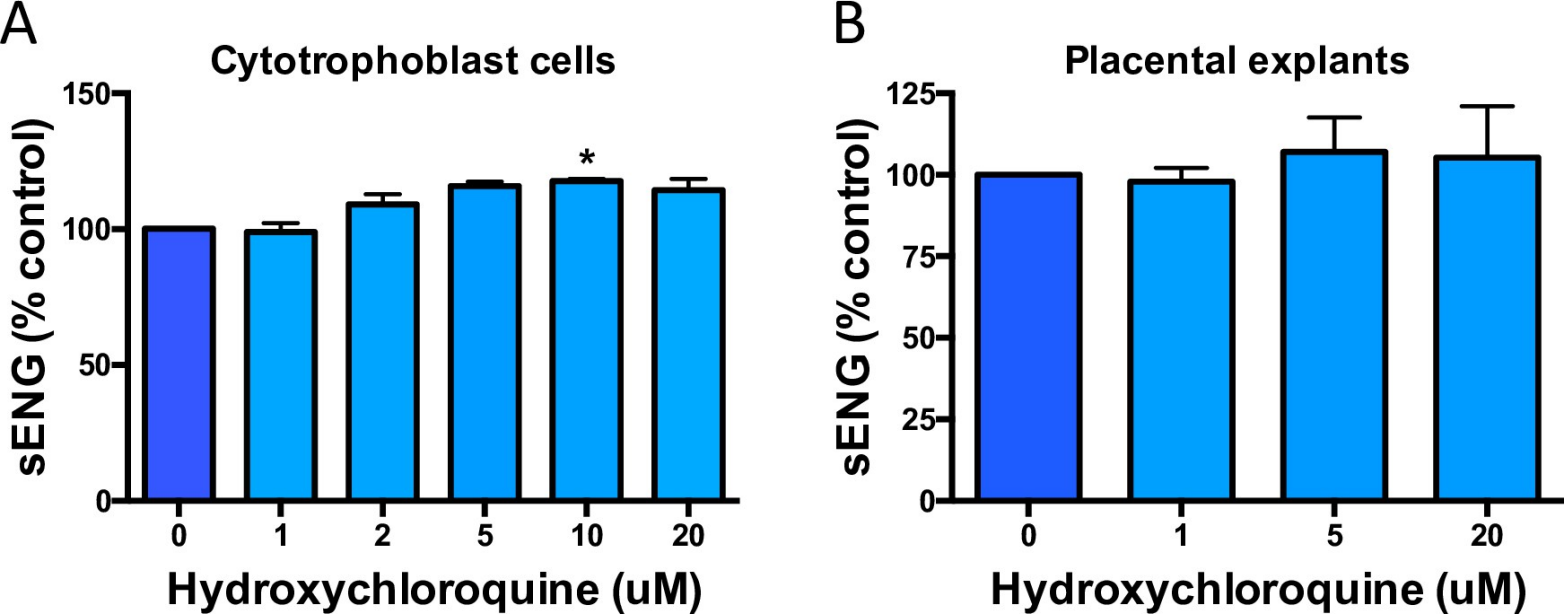
The effects of hydroxychloroquine on sENG in cytotrophoblasts and explants of preeclamptic placentas. Increasing doses of hydroxychloroquine demonstrated a modest increase the secretion of sENG in cytotrophoblasts (a) with a similar but insignificant trend observed in explants (b), compared to untreated controls. Data are means ± SEM of minimum three independent experiments. * p<0.05, (Friedman test).

Next, we examined another anti-angiogenic factor associated with preeclampsia, sENG. Endoglin is a membrane bound glycoprotein, cleaved to a soluble form sENG, which binds to and inhibits pro-angiogenic factors in circulation. Endoglin cleavage involves matrix metalloproteases (MMPs) and there have been suggestions that hydroxychloroquine may inhibit them [17]. As such, we set out to determine whether treating cytotrophoblasts and explants with hydroxychloroquine can reduce the release of sENG from the cells into conditioned media. At the 10μM dose there was an unexpected significant increase in sENG secretion from cytotrophoblasts, which was not observed in any other doses in either cells or tissue explants (Fig 2A and 2B).

### Effect of hydroxychloroquine on *PlGF* and *VEGF* expression and secretion from HUVECs and expression from placental explants

**Fig 3:**
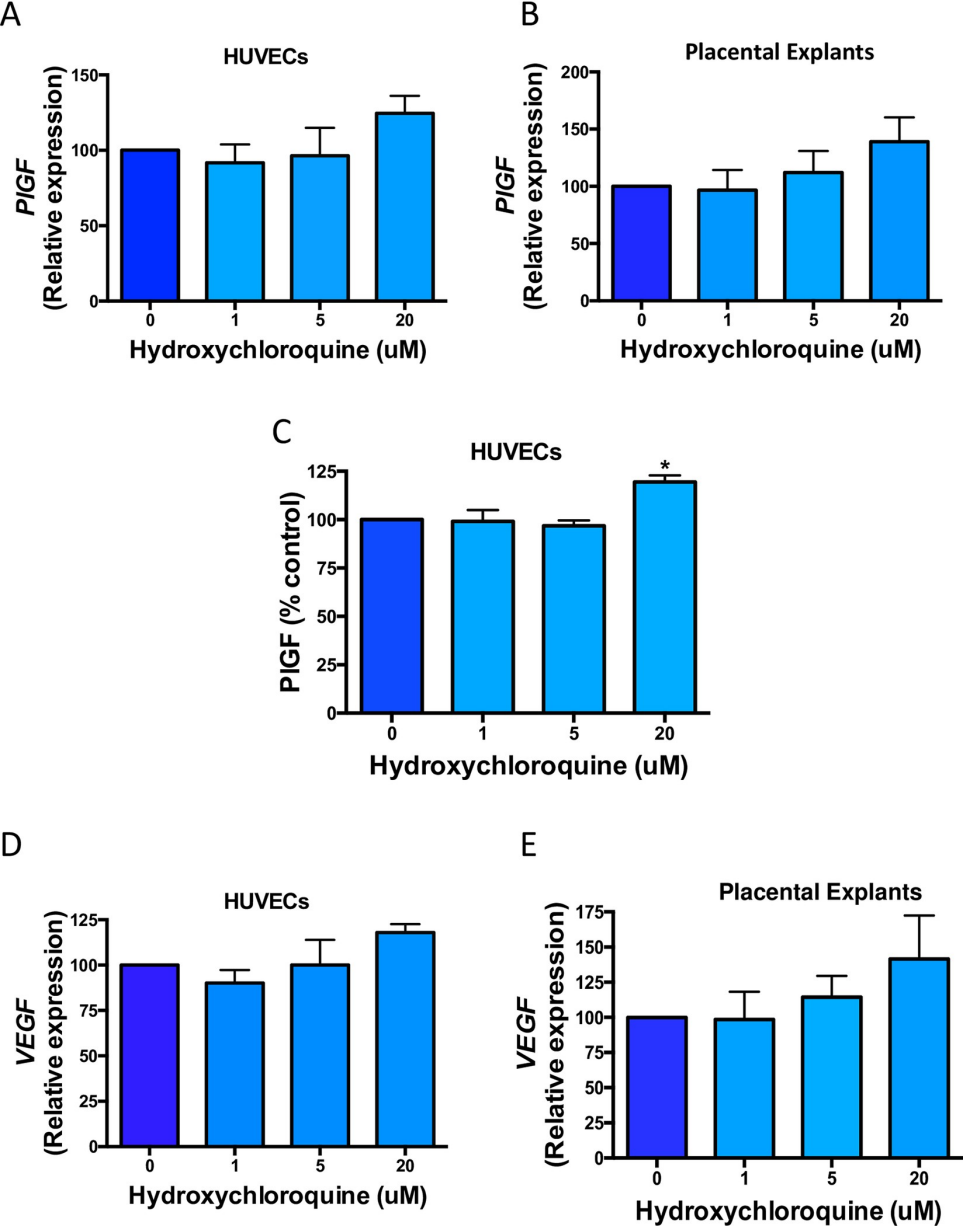
The effects of hydroxychloroquine on *PIGF* and *VEGF* gene expression and secretion in HUVECs and placental explants isolated from normal and preeclamptic placentas, respectively. HUVECs (a) and placental explants (b) treated with the highest dose (20μM) of hydroxychloroquine have on average higher mRNA expression and secretion of *PlGF* from HUVECs (c) is significantly elevated, compared to vehicle treated controls. Higher doses of hydroxychloroquine (5μM and 20μM) also showed an increase in *VEGF* expression in HUVECs (d) and placental explants (e). Data are means ± SEM of minimum three independent experiments. *p<0.05, (Friedman test).

PIGF is a pro-angiogenic factor that is reduced in preeclampsia [18]. Here, we treated HUVECs and placental explants and measured PIGF mRNA expression and secretion into the conditioned media. Highest (20μM) dose of hydroxychloroquine led to an approximate 20% increase in mRNA expression of *PIGF* in both HUVECs and explants (Fig 3A and 3B). This correlates with a significant upregulation in the concentration of PIGF that is secreted from HUVECs, compared to vehicle treated controls (Fig 3C). As such, enhanced secretion of PIGF, coupled with the significant downregulation of sFlt-1 observed in our cytotrophoblasts (Fig 1C), may help equalise the imbalance in angiogenic factors.

VEGF, like PIGF, is a target ligand for vascular endothelial growth factor receptor (VEGFR) and is essential in promoting the proliferation and migration of endothelial cells during angiogenesis [19]. We examined changes to *VEGF* in HUVECs and explants under drug treatment and found a dose dependant increase in gene expression in these cultures (Fig 3D, 3E).

### Effect of hydroxychloroquine on endothelial cell dysfunction markers and functional outcomes in HUVECs

**Fig 4:**
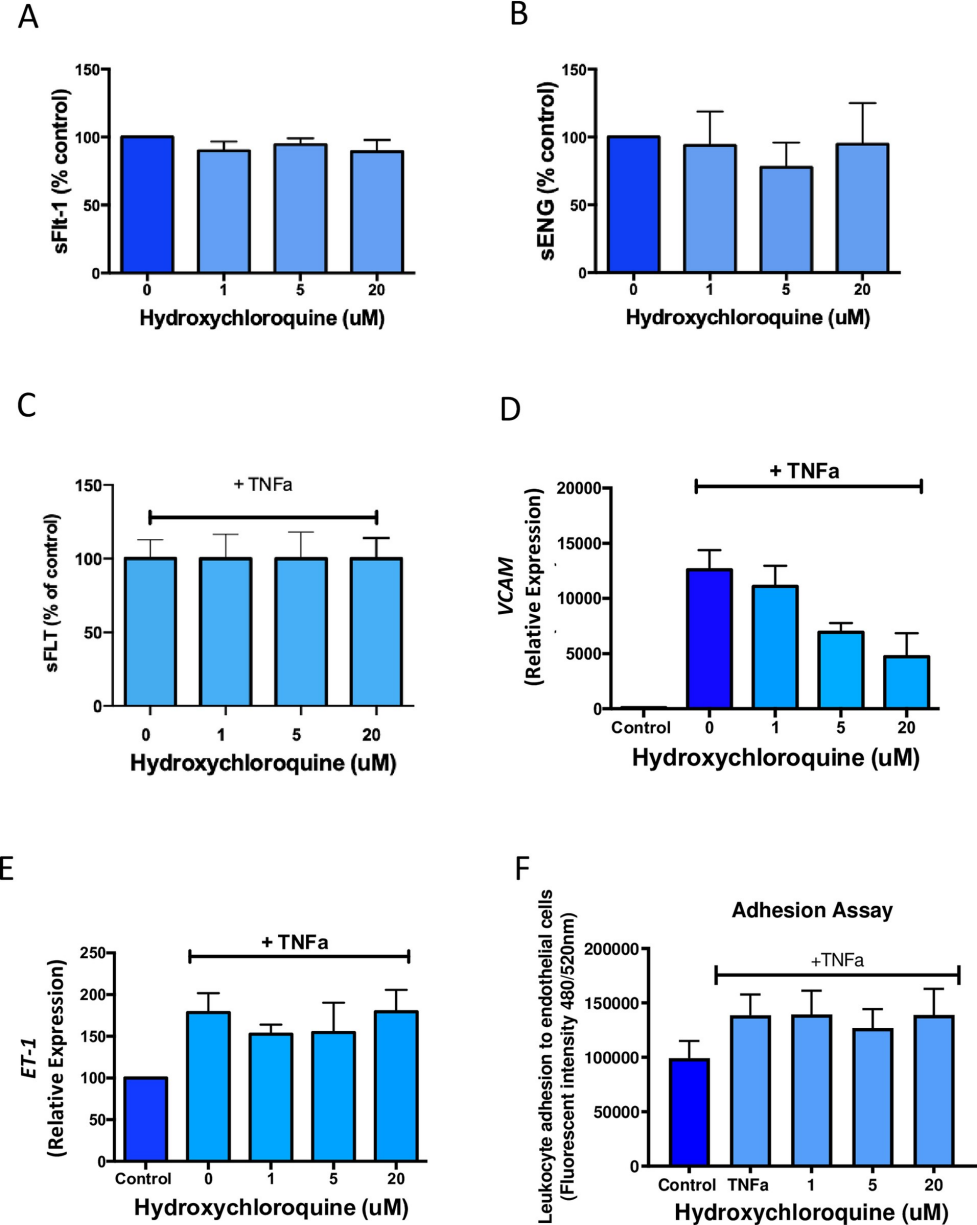
The effects of hydroxychloroquine on HUVEC secretion of sFlt-1 and sENG and endothelial cell dysfunction gene markers. Hydroxychloroquine treatment of HUVECs did not change sFlt-1 (a) or sENG (b) secretion. Hydroxychloroquine did not reduce sFlt-1 secretion in the presence of TNFα. TNFα stimulation significantly increases *VCAM* (d) and *ET1* (e) expression in HUVECs, compared to untreated control cells. Hydroxychloroquine treatment appeared to lower *VCAM* expression, in a non-significant dose dependent manner (d), however, *ET1* levels remain unchanged (e). Adhesion of monocytes to HUVECs increases under TNFα stimulation and hydroxychloroquine treatment does not reliably reverse this outcome (f). Data are means ± SEM of minimum three independent experiments.

We treated HUVECs with increasing doses of hydroxychloroquine and showed there was no change in sFlt-1 (Fig 4A) and sENG (Fig 4B) secretion. Furthermore we treated HUVECs with TNFa, a cytokine known to be increased in preeclampsia, and demonstrated no change to sFlt-1 secretion (Fig 4C).

Disruption to vascular homeostasis is a key abnormality in preeclampsia. ET1 and VCAM are markers of endothelial dysfunction, expression of both can be induced by the pro-inflammatory cytokine TNFα [20]. ET1 is a potent vasoconstrictor that incites reactive oxygen species production and inflammatory signalling in endothelial cells [21]. As such, we used these dysfunction markers to investigate whether hydroxychloroquine could reverse or suppress their expression with TNFα treatment of primary HUVECs.

As expected, expression of *VCAM* and *ET1* more than doubled in vehicle control cells after TNFα stimulation, compared to unstimulated cells (Fig 4D). The doses of hydroxychloroquine used in this study were not adequate in overcoming the significant upregulation of *ET1* in HUVECs (Fig 4B). In contrast, addition of hydroxychloroquine (1–20μM) led to a decreasing trend in *VCAM* expression (Fig 4E), compared to vehicle control treated cells. VCAM is a key component for leukocyte attachment and extravasation through to the sites of inflammation, this can exacerbate endothelial dysfunction and amplify injury to the maternal vessels [22]. Therefore, we set out to assess whether the downregulation in *VCAM* expression by hydroxychloroquine treatment could also functionally reduce the adhesion of leukocytes to inflamed endothelial cells. As such, we performed monocyte adhesion assays, whereby HUVECS were cultured in the presence of TNFα and fluorescently stained PBMCs were then added to the cultures to determine level of cell-cell adhesion. We demonstrated that TNFα induced greater level of adhesion between PMBCs to HUVECs (Fig 4F). Overall, hydroxychloroquine treatment could not overcome this effect (Fig 4F).

## Discussion

Preeclampsia is a multi-systemic vascular disorder that is associated with excess release of anti-angiogenic factors from the placenta that cause systemic maternal endothelial dysfunction [23]. It can have chronic and severe implications for the mother and infant. Currently, the only effective treatment is delivery to minimise complications. The exact mechanism and cause of preeclampsia remains to be elucidated, however there is deregulation in the interplay between immune and vascular components in the placenta, with auto-immune conditions being a known risk-factor [24]. In rheumatic and auto-immune conditions hydroxychloroquine is reported to reduce incidences of preeclampsia and the rate of associated complications, like intrauterine growth restriction (IUGR) and preterm births in these patients [8, 25, 26]. Because of these reports and due to its anti-inflammatory and antioxidant properties, hydroxychloroquine has been proposed as a potential prevention and treatment for preeclampsia [8]. To determine its efficacy as a preeclampsia therapy, we investigated the effect of hydroxychloroquine treatment on some of the hallmarks of the condition.

In pregnancy there is a natural fluctuation between pro-angiogenic factors during early gestation and the steady fall of these factors coinciding with a rise in anti-angiogenic molecules toward the end of pregnancy [19]. The imbalance between pro and anti-angiogenic factors is a defining feature of preeclampsia, and it precedes the clinical presentation of the disease. We found hydroxychloroquine reduced *sFlt-1 e15a* and *i13* variant isoform expression and total protein secretion from cytotrophoblasts. In contrast, hydroxychloroquine had a non-significant effect on the secretion of sFlt-1 from HUVECs and placental explants, under normal culture conditions. Previously, hydroxychloroquine failed to overcome significant upregulation in sFlt-1 and sENG expression in hypoxic placental explants [9]. However, in these experiments, only a single non-cytotoxic dose (1μg/mL) of hydroxychloroquine was used to treat explants for 24 hours. The discrepancy in findings between cytotrophoblasts and explants is likely due to experimental conditions and heterogeneity of cells in the explants. Drug response, in organotypic cultures like explants, may be delayed and require higher doses or longer durations to produce the same effect as monolayer cultures.

We detected a slight increase in *PIGF* and *VEGF* mRNA expression in placental explant tissue and HUVECs, along with a significant increase in PlGF secretion from HUVECs. Perhaps hydroxychloroquine might be reducing the preeclampsia risk in patients with autoimmune conditions by regulating angiogenesis modulators [8, 25, 26]. Changes to PlGF and sFLt-1 levels may have secondary effects on endothelial cell function. This is because sFlt-1 mediated inhibition of pro-angiogenic factors and VEGFR signalling, not only disrupts angiogenesis but it also alters cellular metabolism. It promotes a glycolytic switch with reduced mitochondrial respiration and an excess production of reactive oxygen species (ROS), prompting oxidative stress in endothelial cells [27, 28]. Additionally, the implications of sFlt-1 are not only confined to the placenta, but these effects can also be observed in organs with high VEGF expression, like the kidneys and liver, possibly contributing to clinical presentation of systemic complications of preeclampsia [29, 30]. Oxidative injury caused by sFlt-1 is also reported to sensitise endothelial cells to vasoconstrictors, ultimately causing hypertension [31].

ET1 is one such vasoconstrictor, which stimulates ROS mediated endothelial cell damage driving the expression of adhesion markers, like VCAM. Both factors are upregulated in preeclampsia patients [21, 32, 33]. In our experiments, TNFα induced damage to endothelial cells was only partly reversed *VCAM* mRNA expression by hydroxychloroquine treatment, while, *ET1* mRNA transcripts remained unchanged. Additionally, we did not see a reduction in monocyte adhesion to endothelial cells in the presence of hydroxychloroquine. Interestingly, hydroxychloroquine had previously been shown to rescue tight junction loss in human umbilical vein derived endothelial cells (HUVECs), mediated by tumour necrosis factor-α (TNFα) and preeclampsia sera treatments, *in vitro* [9]. Therefore, there may be elements of endothelial dysfunction in preeclampsia that hydroxychloroquine can reverse.

There has been interest in repurposing medications safe in pregnancy as candidate preventions and treatments for preeclampsia. We have previously demonstrated metformin [34], sulfasalazine [35] and proton pump inhibitors [20] all reduce antiangiogenic factor secretion and rescue endothelial dysfunction in primary placental tissues and cells and endothelial cells respectively. We now have promising clinical trial data demonstrating metformin may be a treatment for preterm preeclampsia [36]. We have also demonstrated that small molecules may have an additive effect when used in combination [37, 38]. Given hydroxychloroquine did not rescue endothelial dysfunction in our assays, perhaps it may have be additive or synergistic when used in combination with another candidate therapy capable of reducing endothelial dysfunction.

In conclusion, hydroxychloroquine reduces anti-angiogenic sFlt-1 production, but does not reduce endothelial dysfunction. It may have potential as a therapy or prevention if used in combination with medications that mitigate endothelial dysfunction.

## Supporting information

S1 File (PZFX)

S2 File (PZFX)
